# Supplementary material for: Facile Attachment of Halides and Pseudohalides to Dodecaborate(2-) via Pd-catalyzed Cross-Coupling
Source: Molecules. 2023 Apr 5;28(7):3245. doi: 10.3390/molecules28073245 (PMC10096879; doi:10.3390/molecules28073245)
Supplement: Supplementary file 1 [file molecules-28-03245-s001.zip › molecules-2310774-supplementary.pdf]

Facile Attachment of Halides and Pseudohalides to Dodecaborate(2-) via Pd-catalyzed Cross-Coupling

Mahmoud K. Al-Joumhawy, Jui-Chi Chang, Fariba Sabzi and Detlef Gabel *

Supporting Materials

Contents

General Information: 1

General Procedure: 2

NMR Spectra: 3

MS spectra 11

General Information:

Reagents and solvents were commercially available and used without further purification. $B_{12}H_{11}I$ was prepared according to the literature procedure with some modification.[1] Tetrabutylammonium bromide, tetrabutylammonium chloride, tetrabutylammonium fluoride trihydrate, copper cyanide, sodium bromide, sodium chloride, tetrabutylammonium azide, sodium azide, Davephos, and $Pd_2(dba)_3$ were purchased from Sigma-Aldrich and were used as received. DMSO, dichloromethane, acetonitrile and silica gel (Grade 60, 230-400 Mesh) were from Carl Roth. Celite (545 filter aid, not acid washed, powder) was from Fisher. All cross-coupling reactions were performed in an oven dried 10ml round bottom flask. Thinlayer chromatography (TLC) AluSil plates were from Macherey-Nagel. TLC samples for borane-containing compounds were stained with 1 wt. % $PdCl_2$ in 6 M HCl and were developed using a heat gun. 11B NMR spectra were recorded on a JEOL 400 MHz spectrometer at 25 °C. Chemical shifts were referenced relative to external BF_3 etherate. MestReNova V10.0.2-15465 S3 software was used to visualize the spectra. Coupling constants (J) are reported in Hertz (Hz). Mass spectra in the negative-ion mode were recorded with a WATERS QTOF Premier spectrometer. An open-vessel microwave oven (CEM Discover, Model 908860, or HNZXIB, Model MCR-3) was used.

1. Al-Joumhawy, M.; Cendoya, P.; Shmalko, A.; Marei, T.; Gabel, D., Improved synthesis of halo- and oxonium derivatives of dodecahydrido-closo-dodecaborate(2-). J. Organomet. Chem. 2021, 949, 121967.

General Procedure:

A dry 10 mL round bottom flask fitted with condenser was charged with 1.0 equiv of $(Bu_4N)_2B_{12}H_{11}I$, $Pd_2(dba)_3$ (5 mol%), Davephos (10 mol%), and (2-4 equiv) of Bu_4NBr or NaBr for synthesis of $(Bu_4N)_2B_{12}H_{11}Br$, Bu_4NCl or NaCl for synthesis of $(Bu_4N)_2B_{12}H_{11}Cl$, Bu_4NN_3 or NaN_3 for synthesis of $(Bu_4N)_2B_{12}H_{11}N_3$, $Bu_4NF.3H_2O$ (4.0 equiv) and KOH (4.0 equiv) for synthesis of $(Bu_4N)_2B_{12}H_{11}OH$, copper(I) cyanide for synthesis of $(Bu_4N)_2B_{12}H_{11}CN$. Subsequently 2.0 mL of anhydrous DMSO was added. The reaction flask was filled with N_2 and connected to a condenser. The round bottom flask was placed in a CEM microwave oven at 150 °C, maximum power 300 W, for 15 mins with stirring (high) or heated in an oil bath at 150 °C for 3-5 hours until the starting $(Bu_4N)_2B_{12}H_{11}I$ was completely consumed as judged by ^{11}B NMR and TLC. The mixture was cooled to room temperature and then filtered through a funnel filled with cotton, celite and filter paper. The resulting solution was concentrated under reduced pressure. The crude product was subjected to silica gel chromatography using a gradient of acetonitrile (0-25%) in DCM.

NMR Spectra:

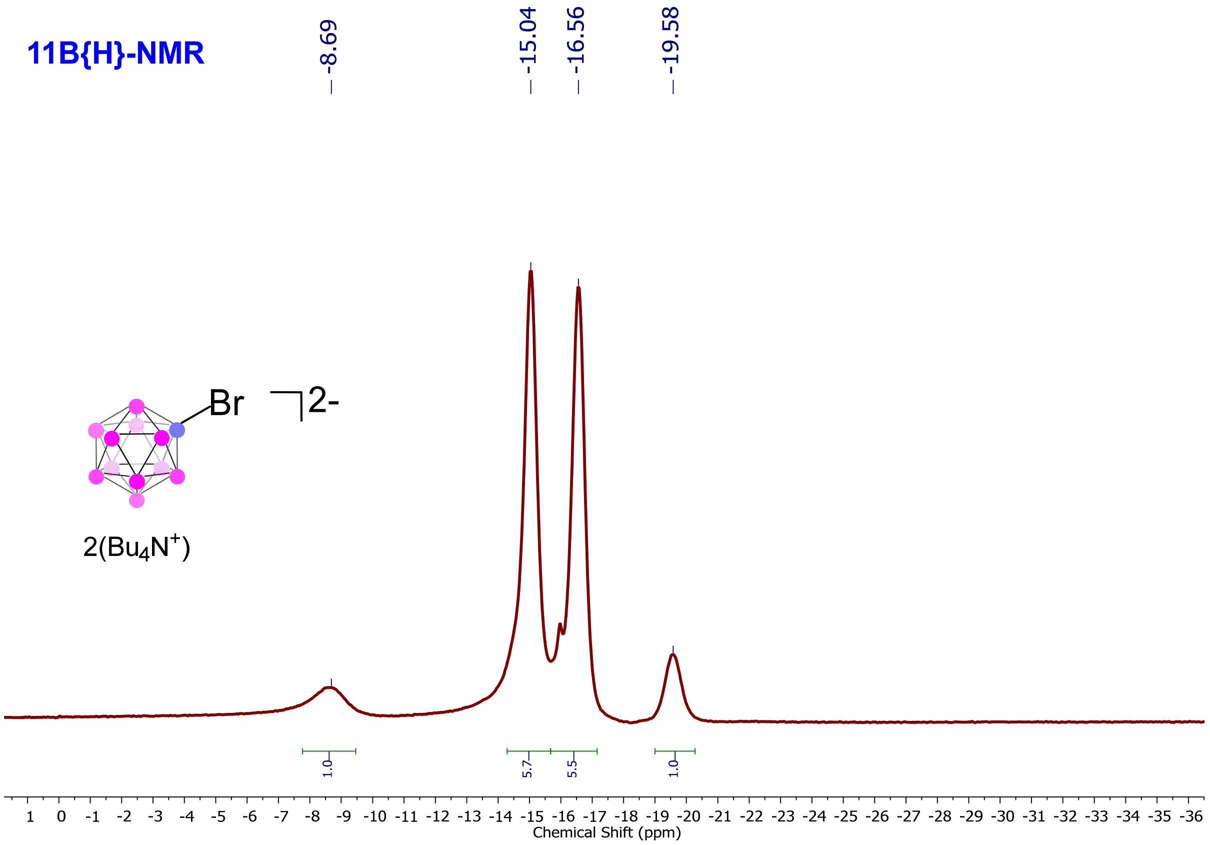

Figure S1. $^{11}B\{H\}$-NMR of $(Bu_4N)_2B_{12}H_{11}Br$.

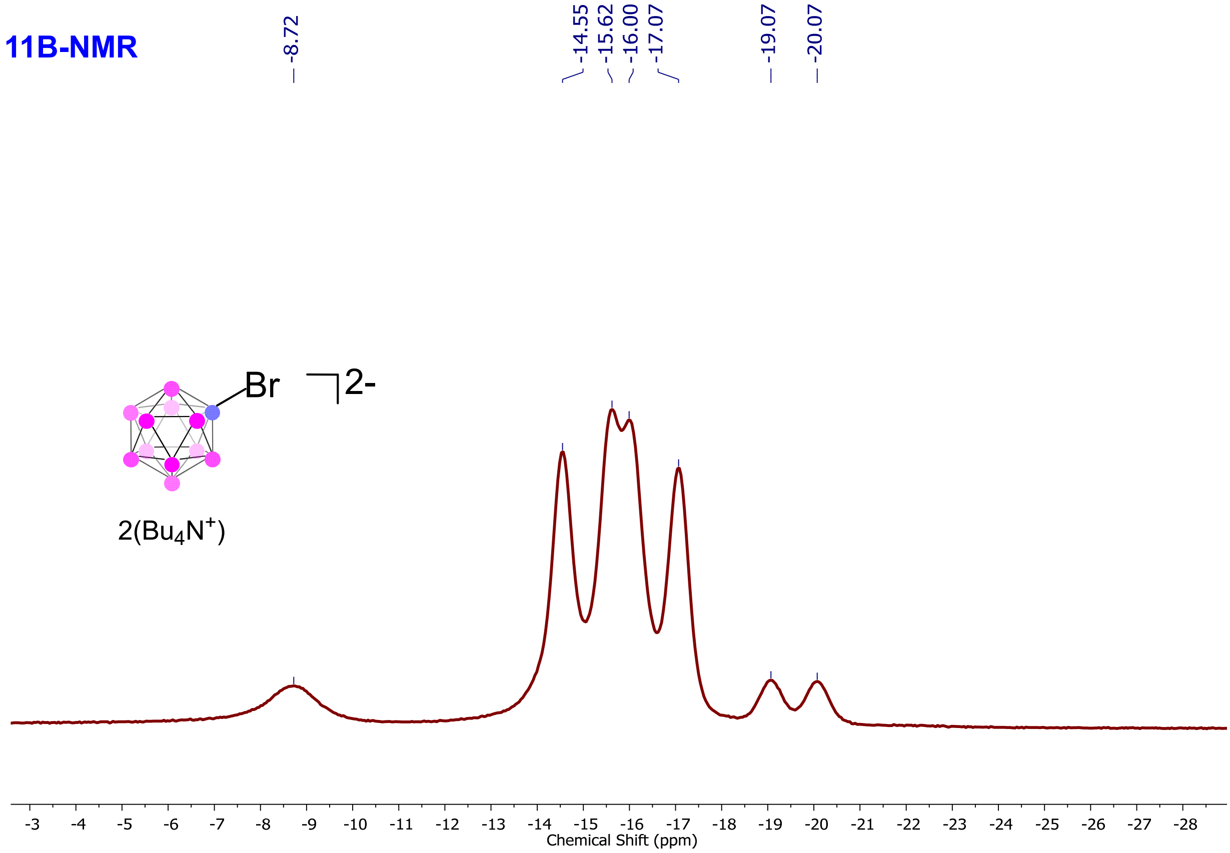

Figure S2. ^{11}B-NMR of $(Bu_4N)_2B_{12}H_{11}Br$.

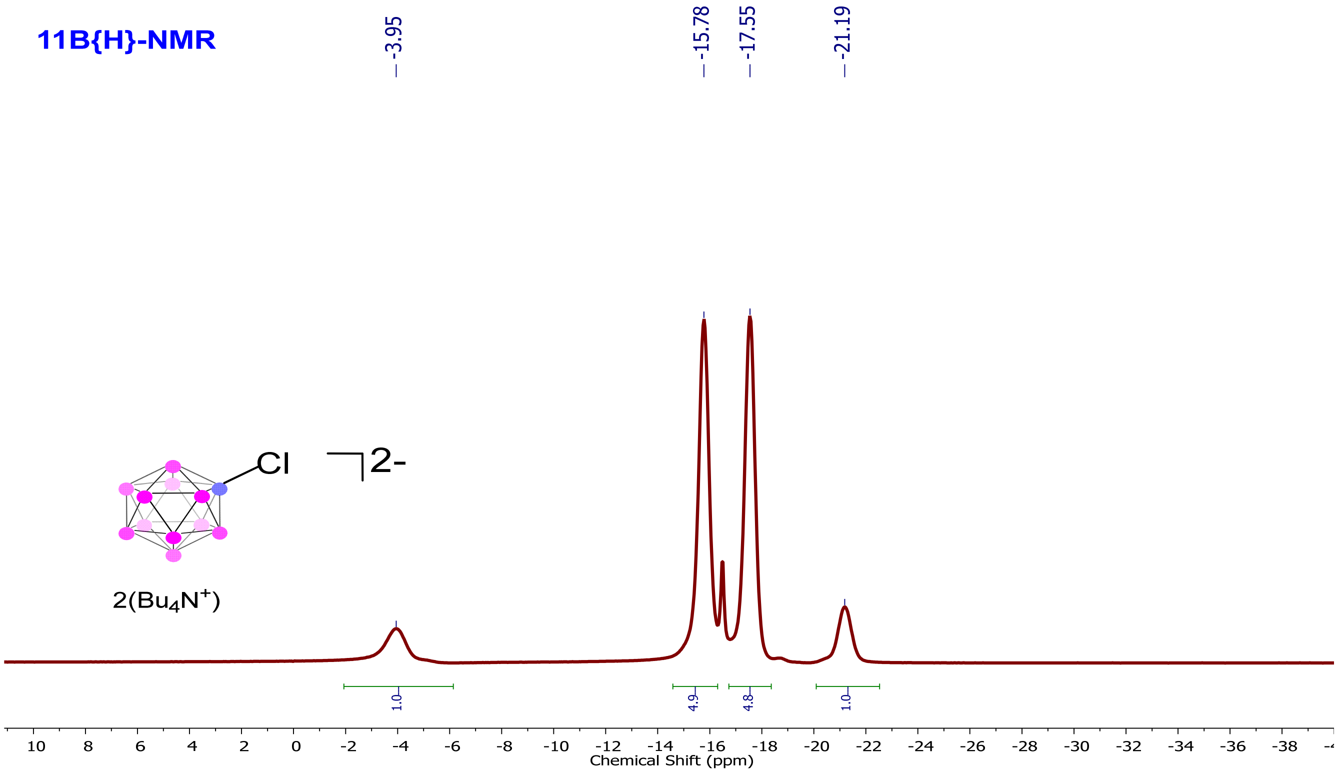

Figure S3. $^{11}B\{H\}$-NMR of $(Bu_4N)_2B_{12}H_{11}Cl$.

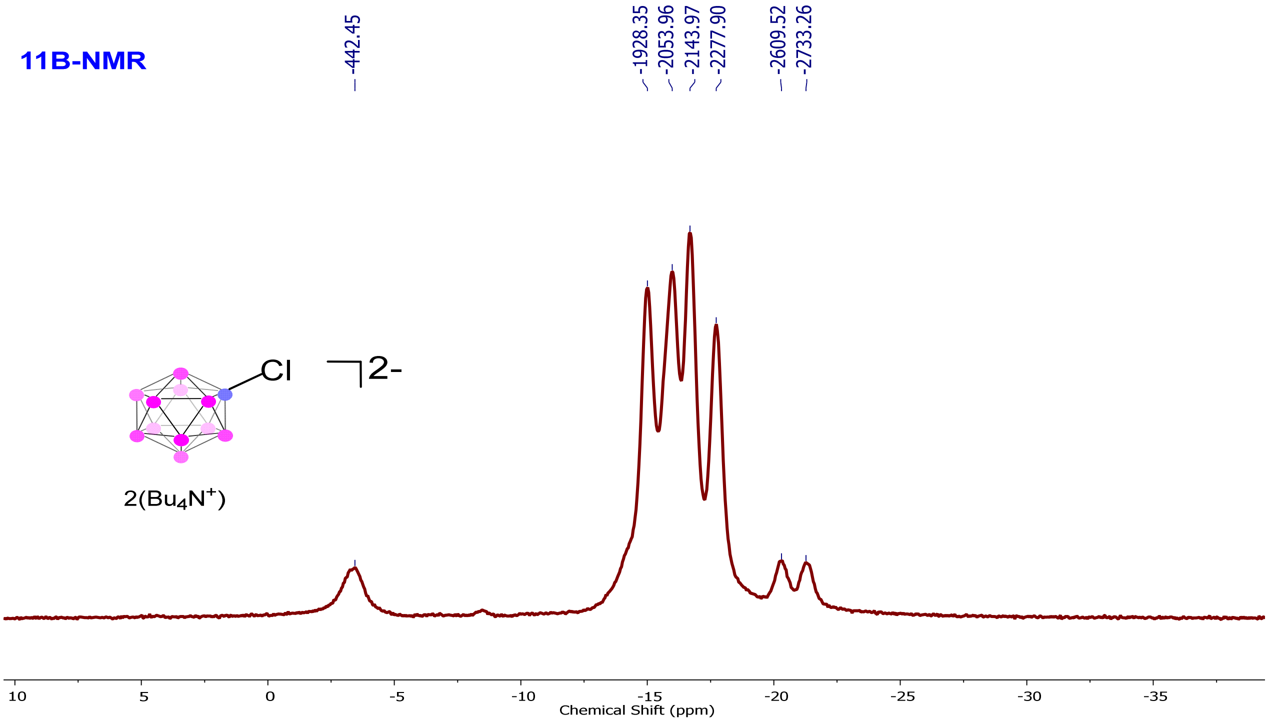

Figure S4. ^{11}B-NMR of $(Bu_4N)_2B_{12}H_{11}Cl$.

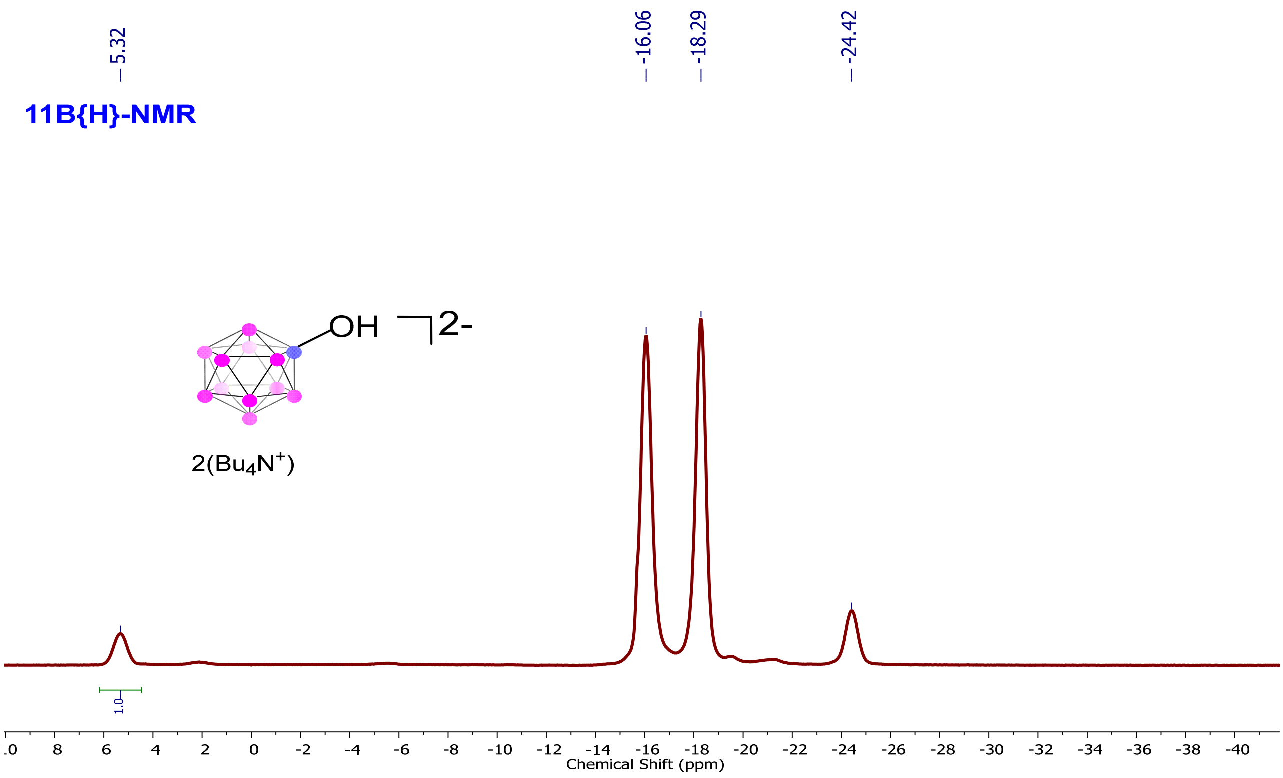

Figure S5. $^{11}B\{H\}$-NMR of $(Bu_4N)_2B_{12}H_{11}OH$.

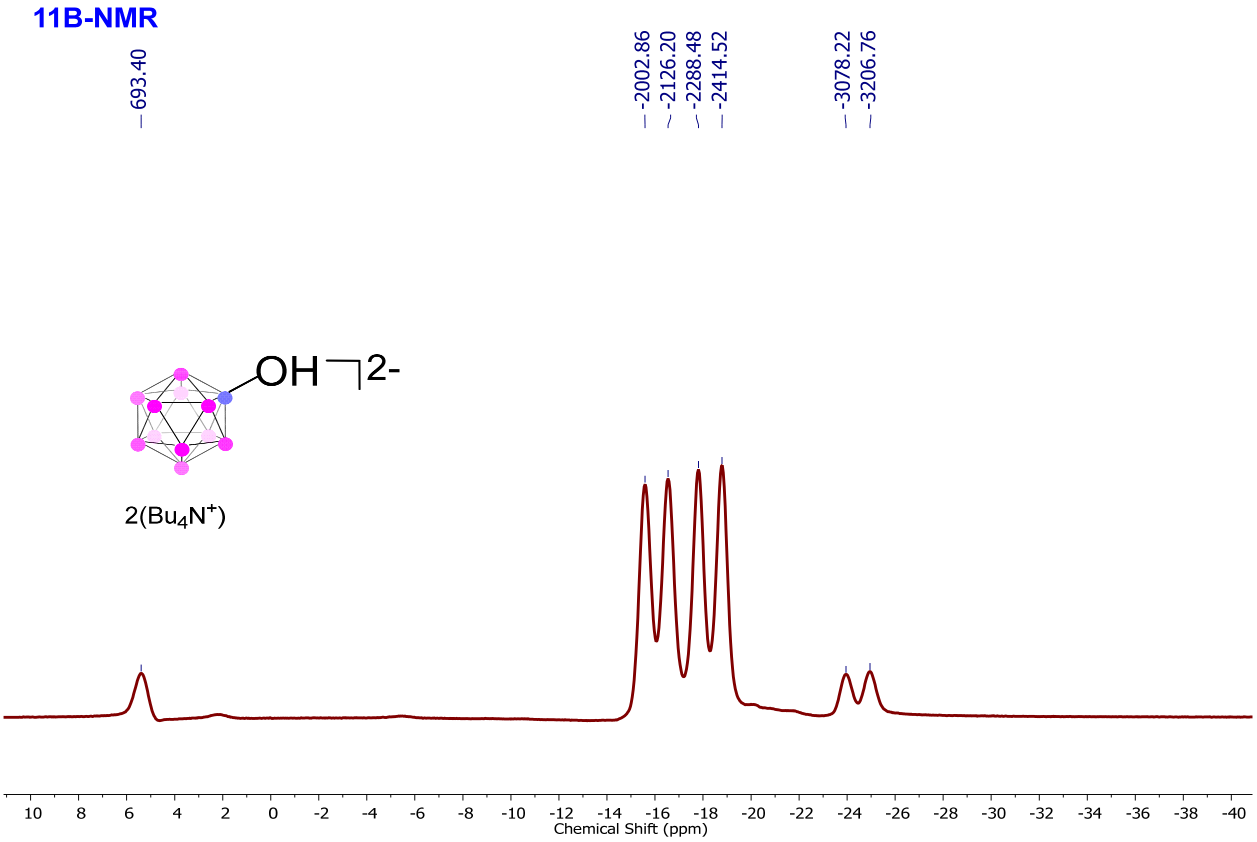

Figure S6. ^{11}B-NMR of $(Bu_4N)_2B_{12}H_{11}OH$.

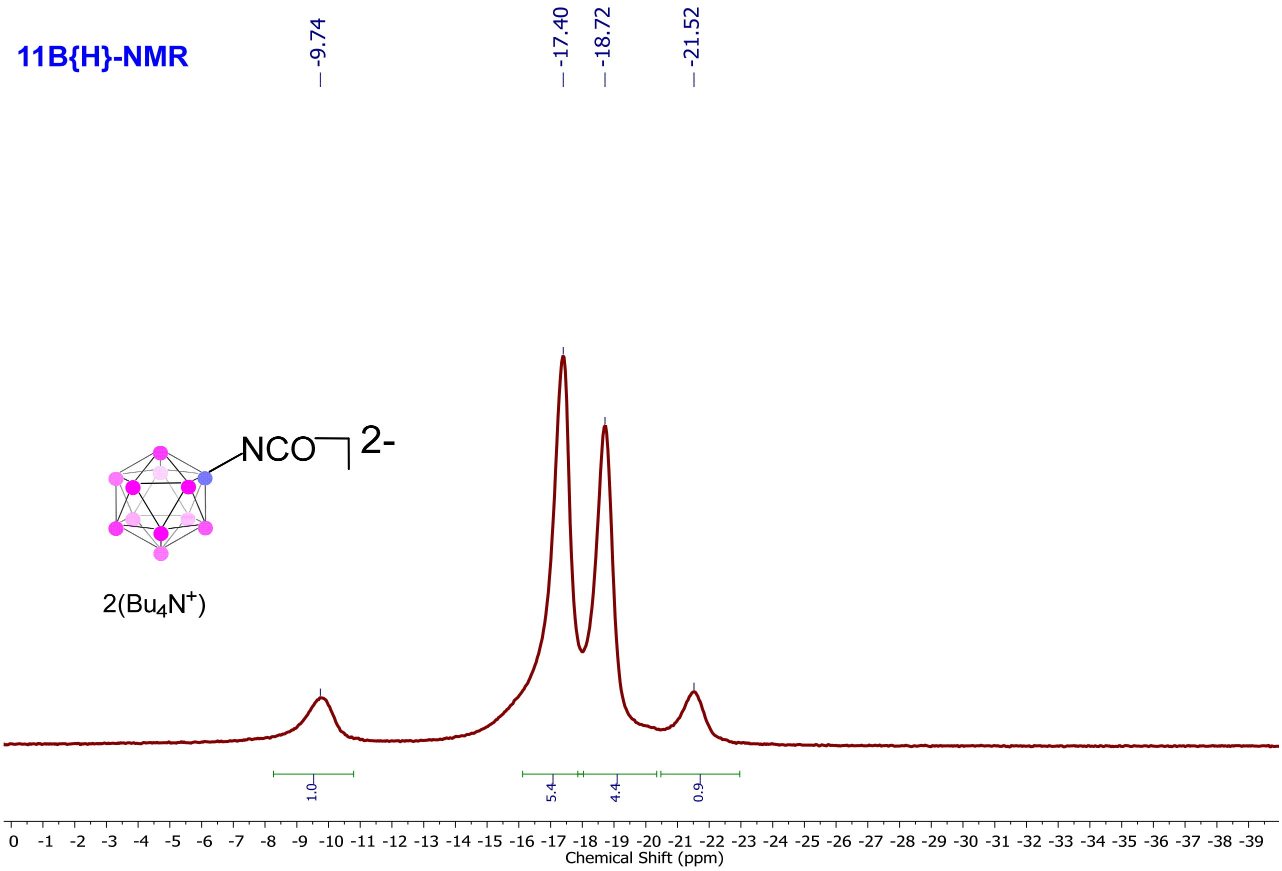

Figure S7. ^{11}B{H}-NMR of $(Bu_4N)_2B_{12}H_{11}NCO$.

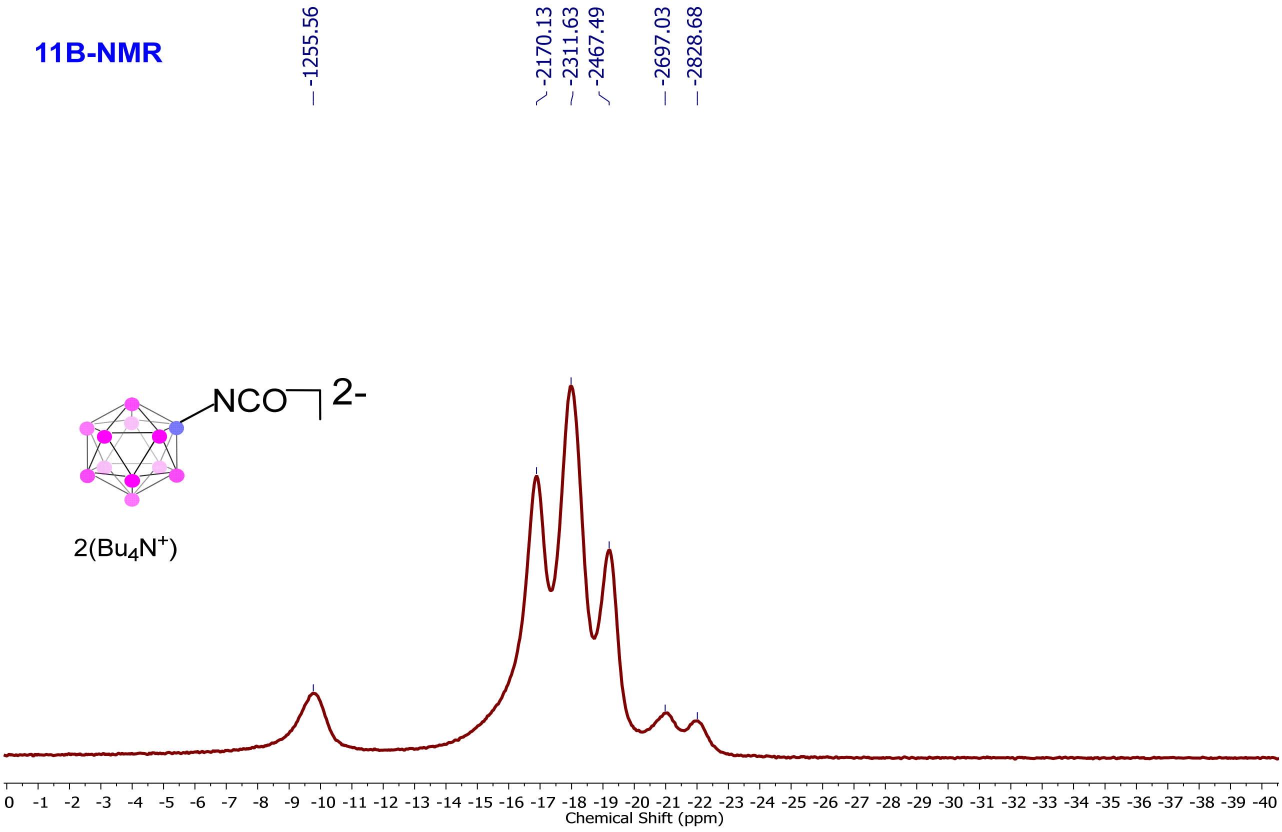

Figure S8. ^{11}B-NMR of $(Bu_4N)_2B_{12}H_{11}NCO$.

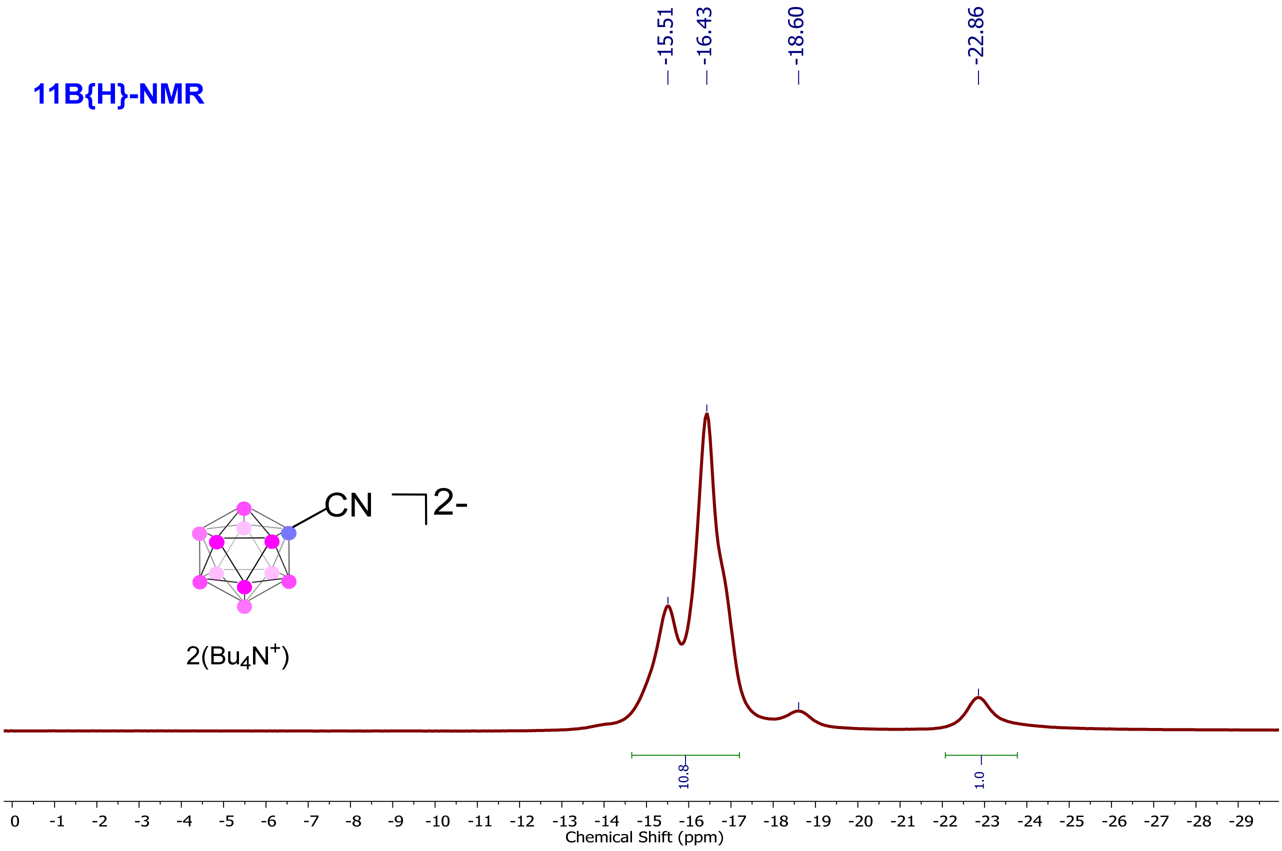

Figure S9. $^{11}B\{H\}$-NMR of $(Bu_4N)_2B_{12}H_{11}CN$.

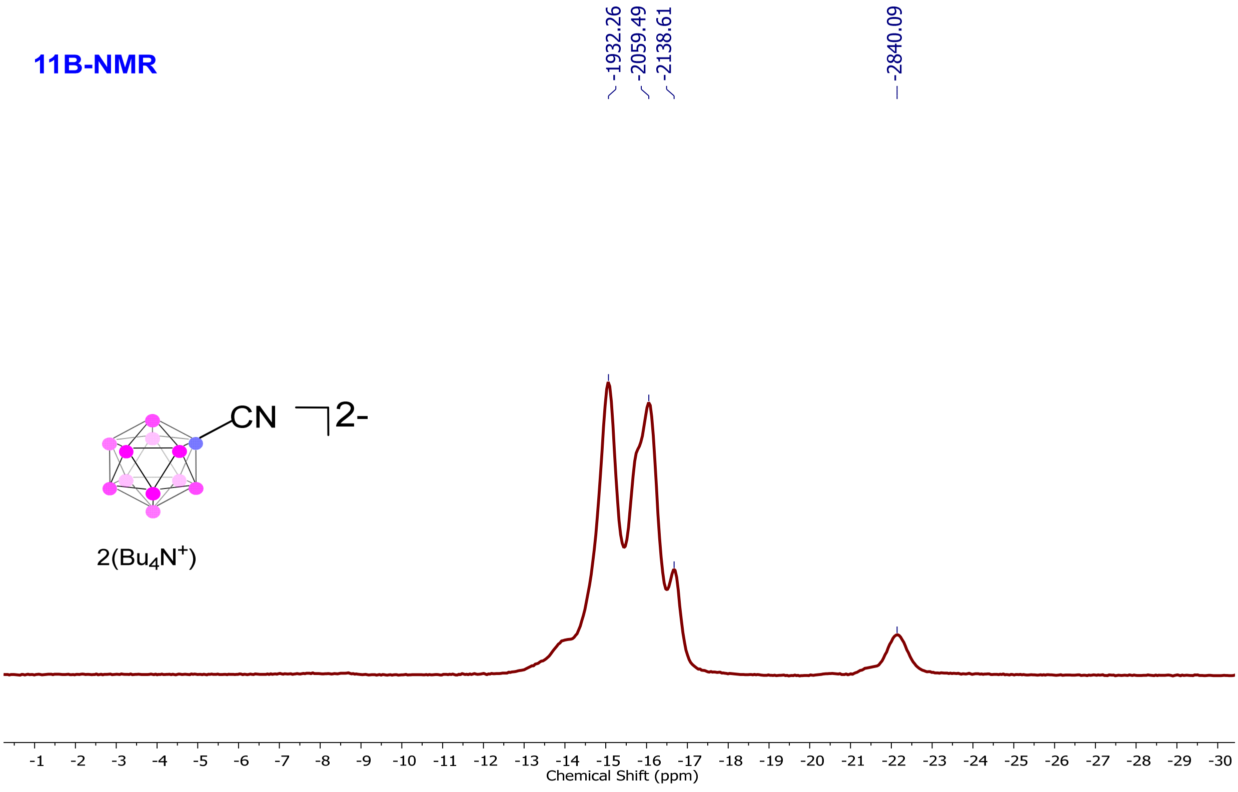

Figure S10. $^{11}B\{H\}$-NMR of $(Bu_4N)_2B_{12}H_{11}CN$.

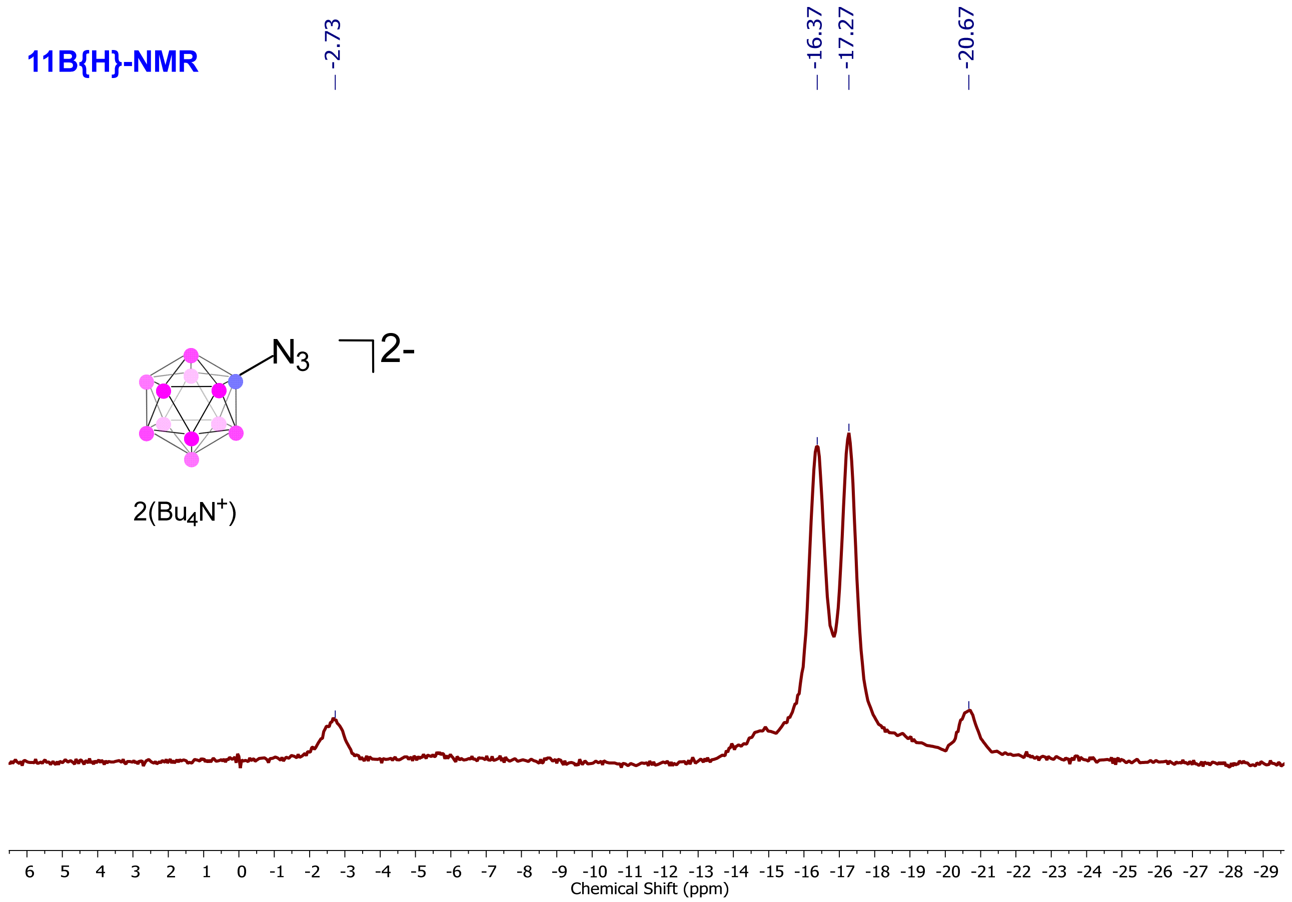

Figure S11. $^{11}B\{H\}$-NMR of $(Bu_4N)_2B_{12}H_{11}N_3$.

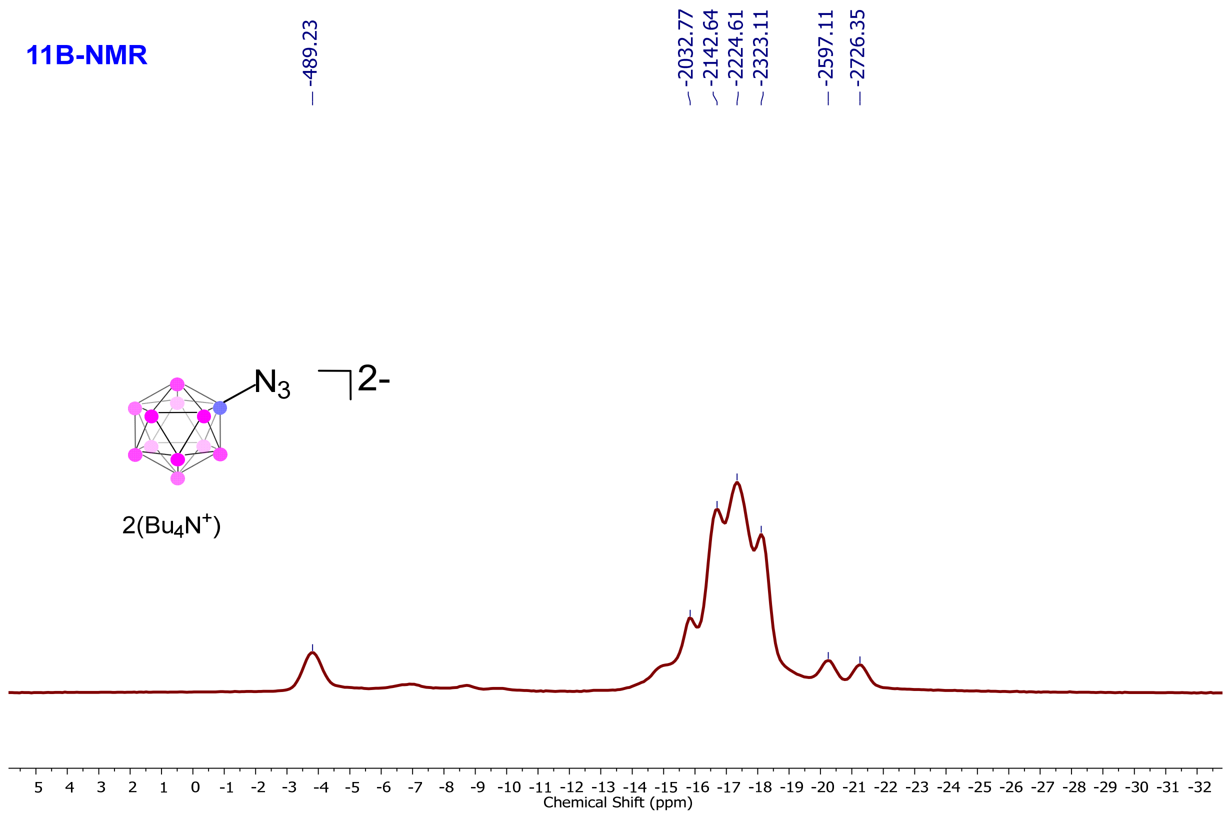

Figure S12. $^{11}B\{H\}$-NMR of $(Bu_4N)_2B_{12}H_{11}N_3$.

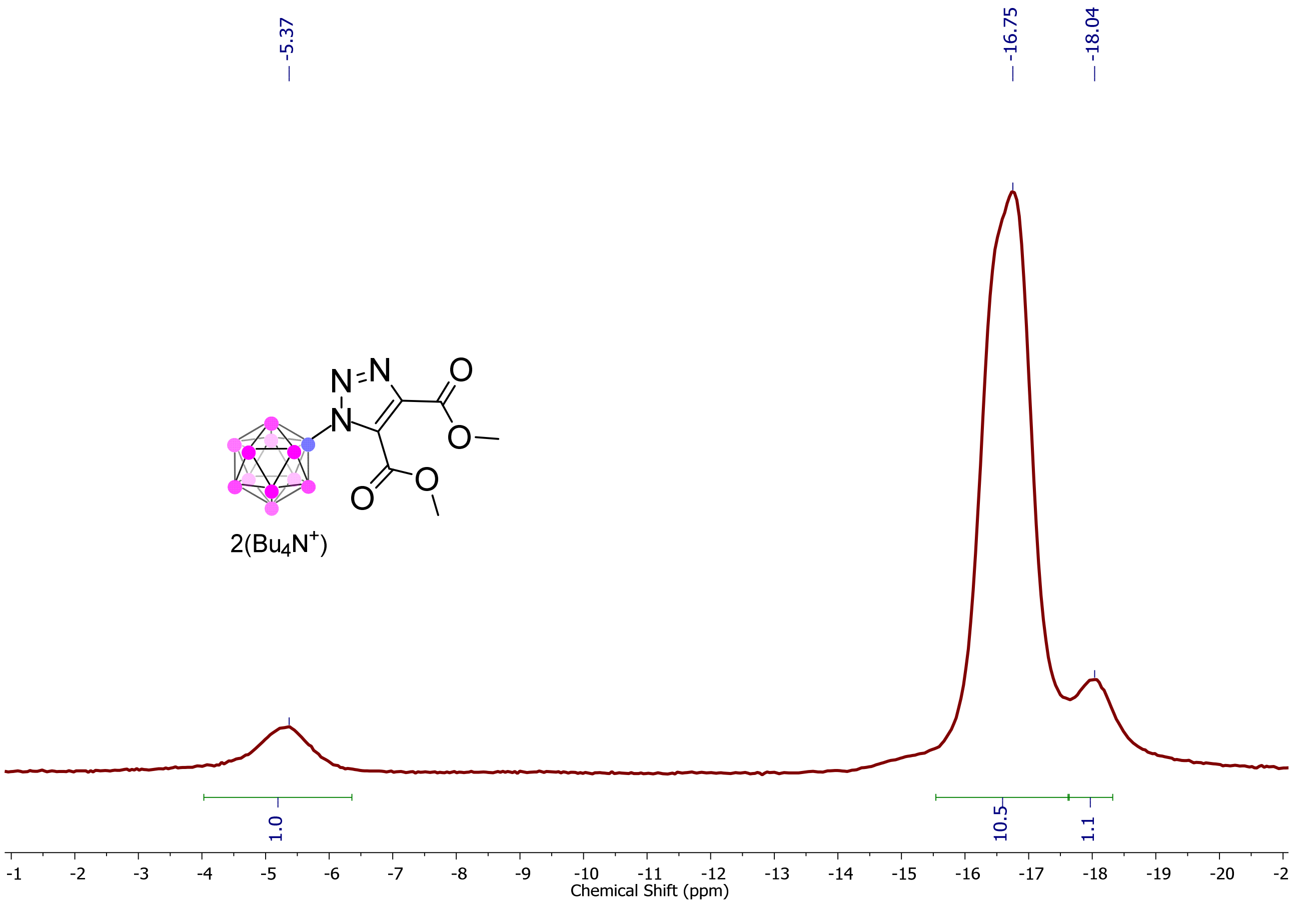

Figure S13. $^{11}B\{H\}$-NMR of $(Bu_4N)_2B_{12}H_{11}$-$C_6H_6O_4$.

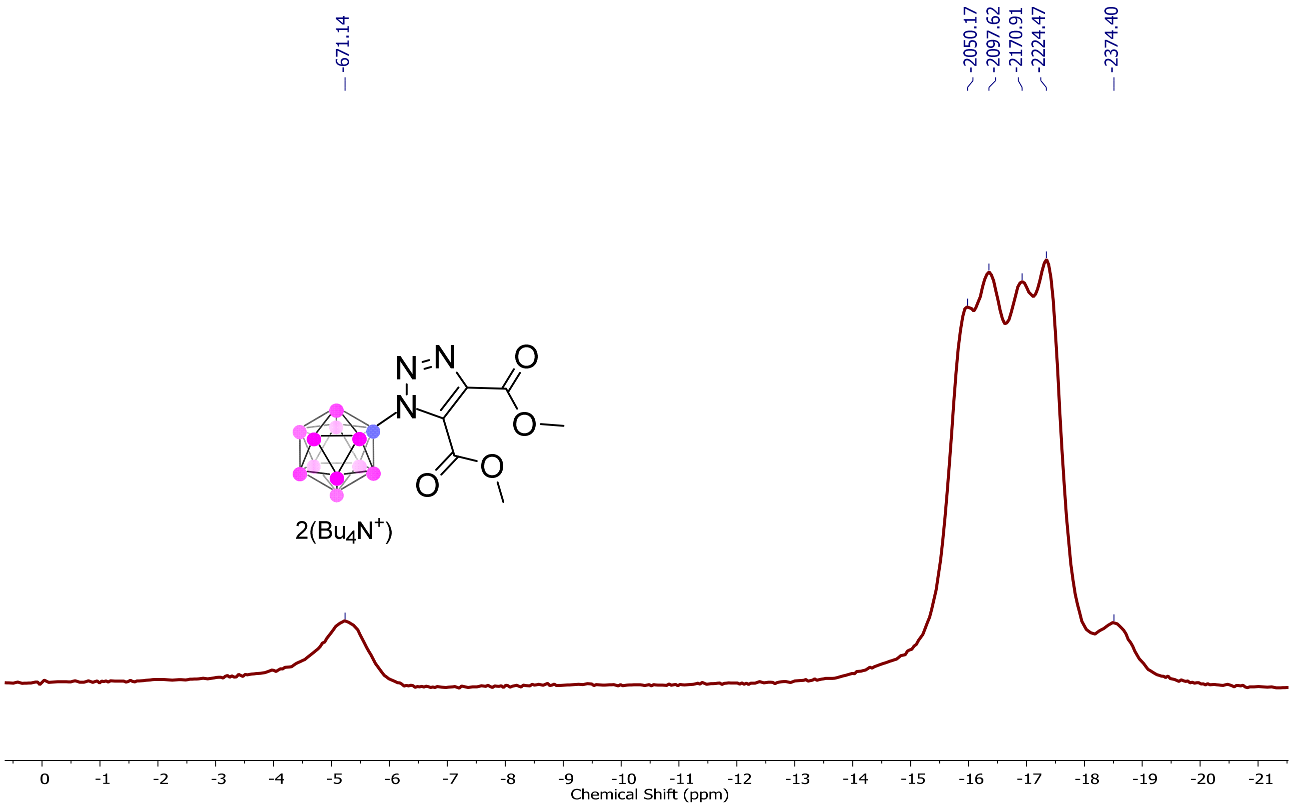

Figure S14. ^{11}B-NMR of $(Bu_4N)_2B_{12}H_{11}$-$C_6H_6O_4$.

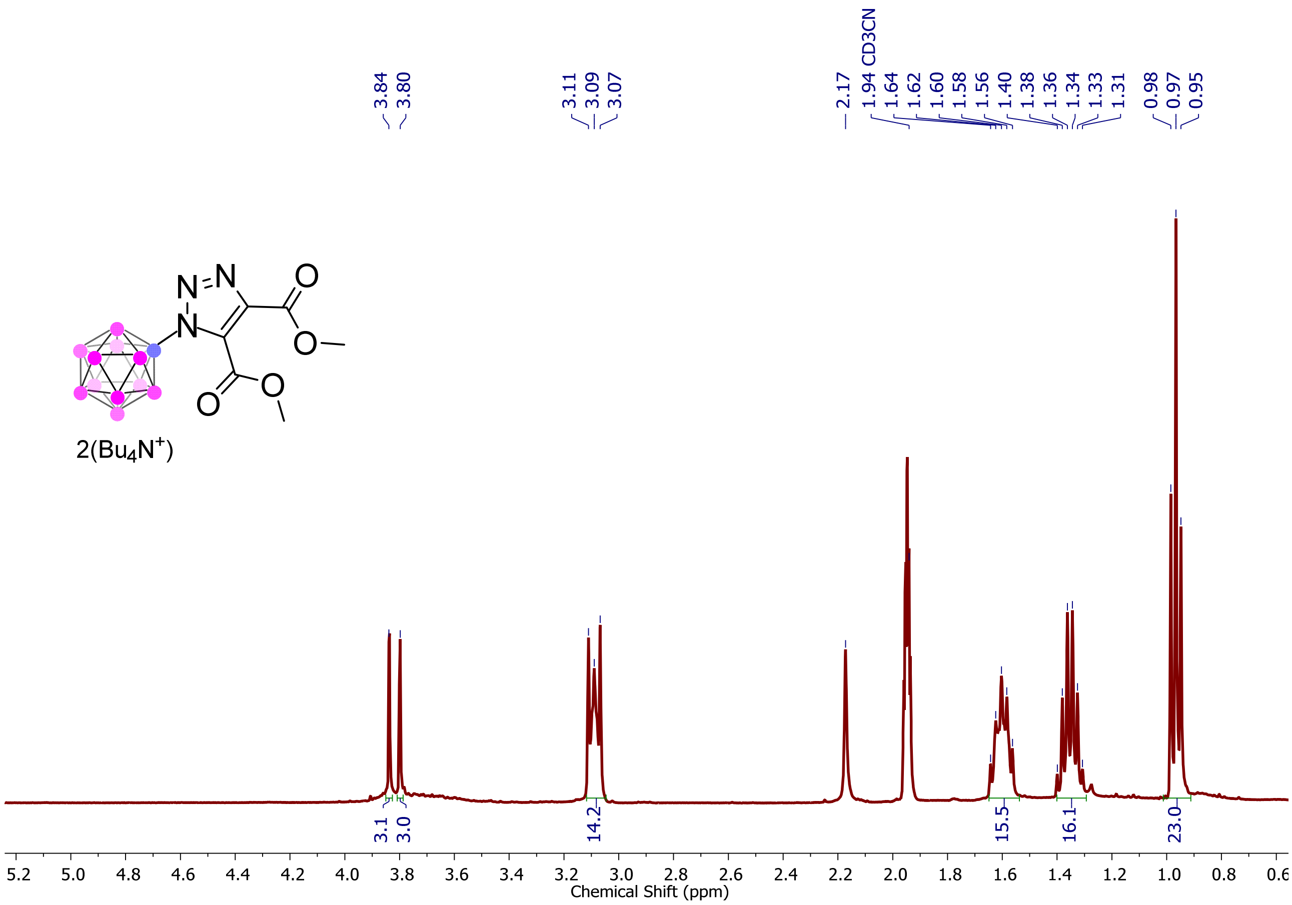

Figure S15. ^{1}H-NMR of the click reaction product.

MS spectra:

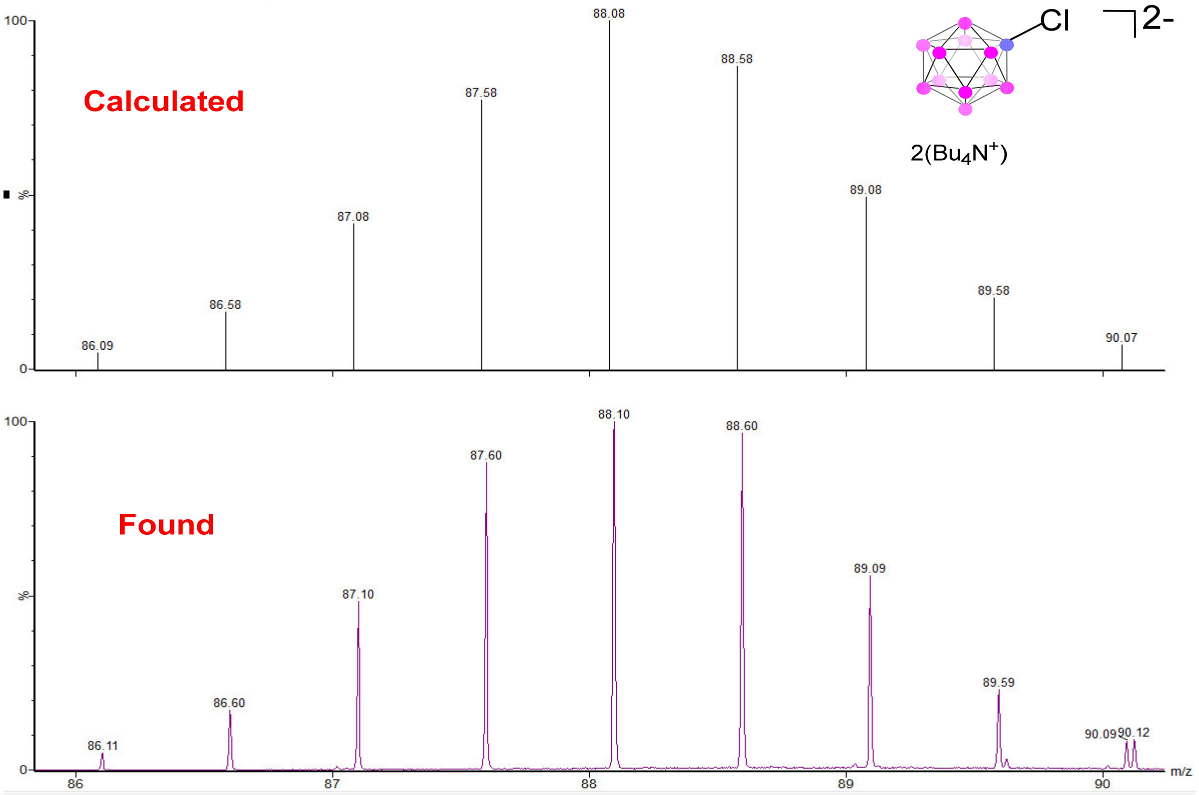

Figure S16. MS spectrum of $[B_{12}H_{11}Cl]^{2-}$.

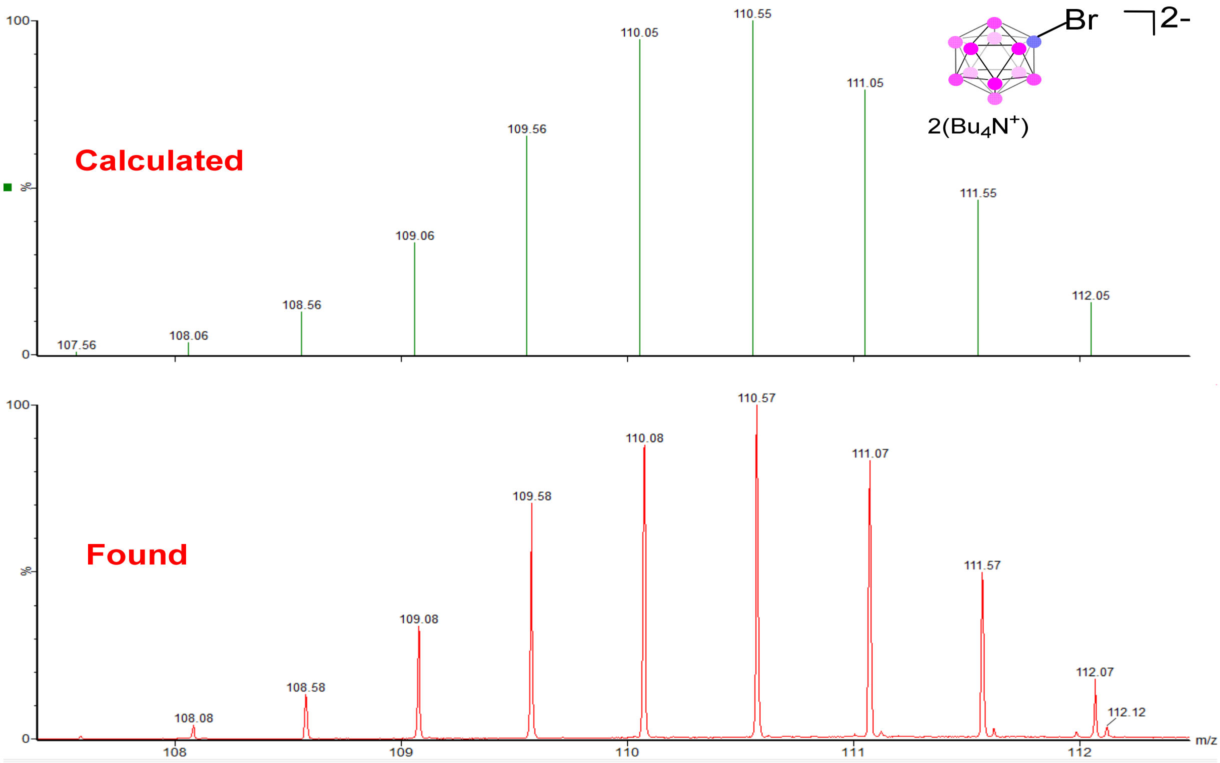

Figure S17. MS spectrum of $[B_{12}H_{11}Br]^{2-}$.

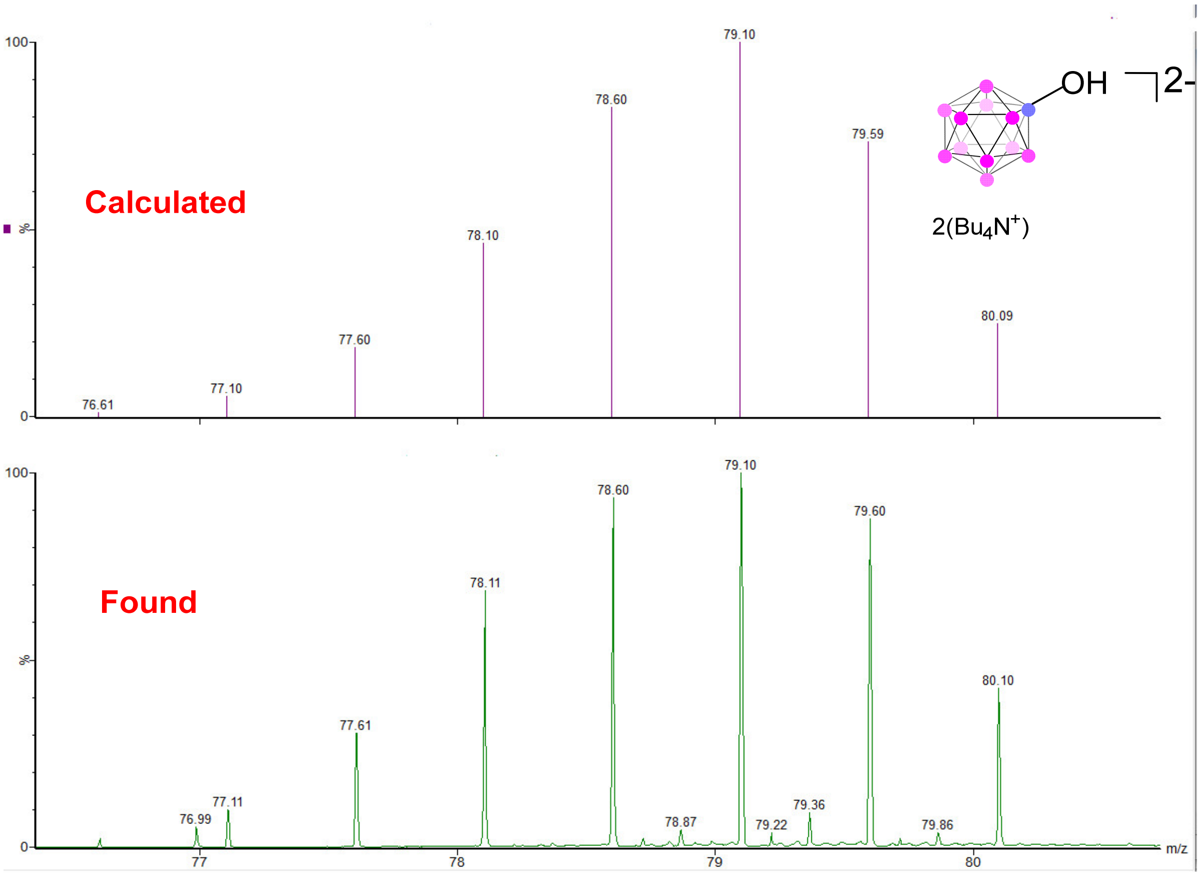

Figure S18. MS spectrum of $[B_{12}H_{11}OH]^{2-}$.

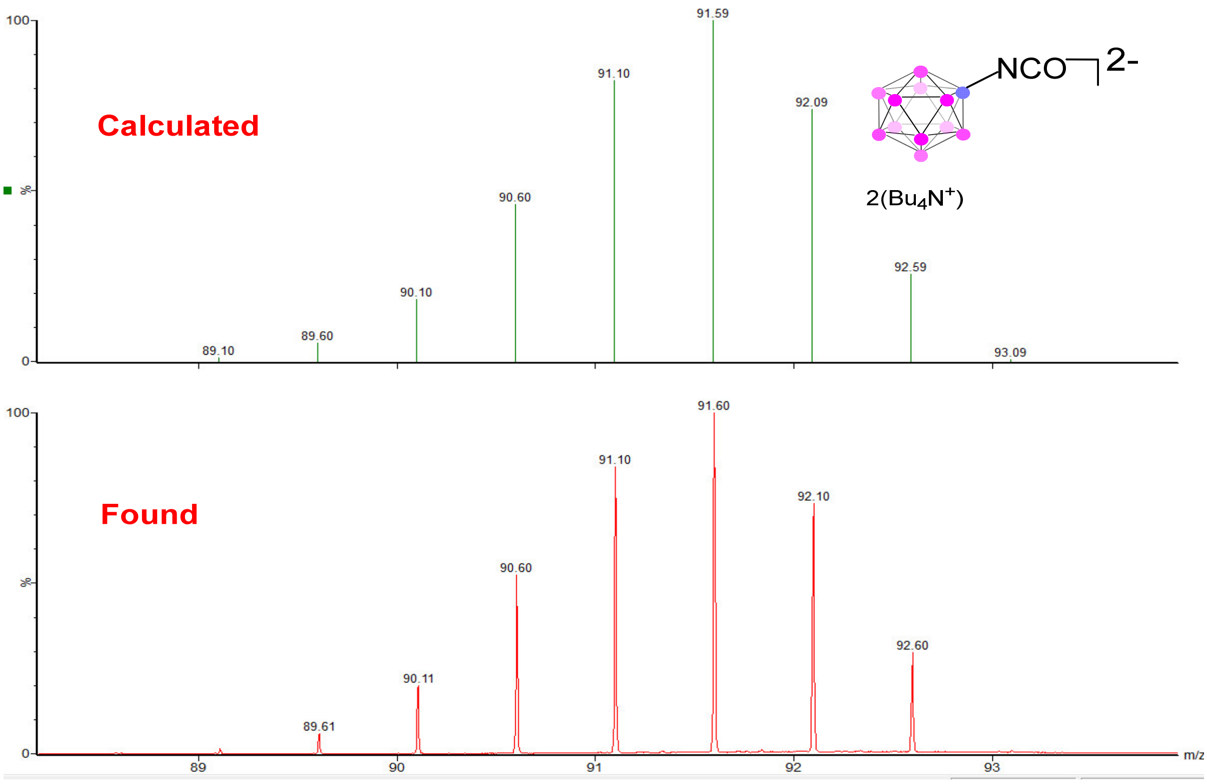

Figure S19. MS spectrum of $[B_{12}H_{11}NCO]^{2-}$.

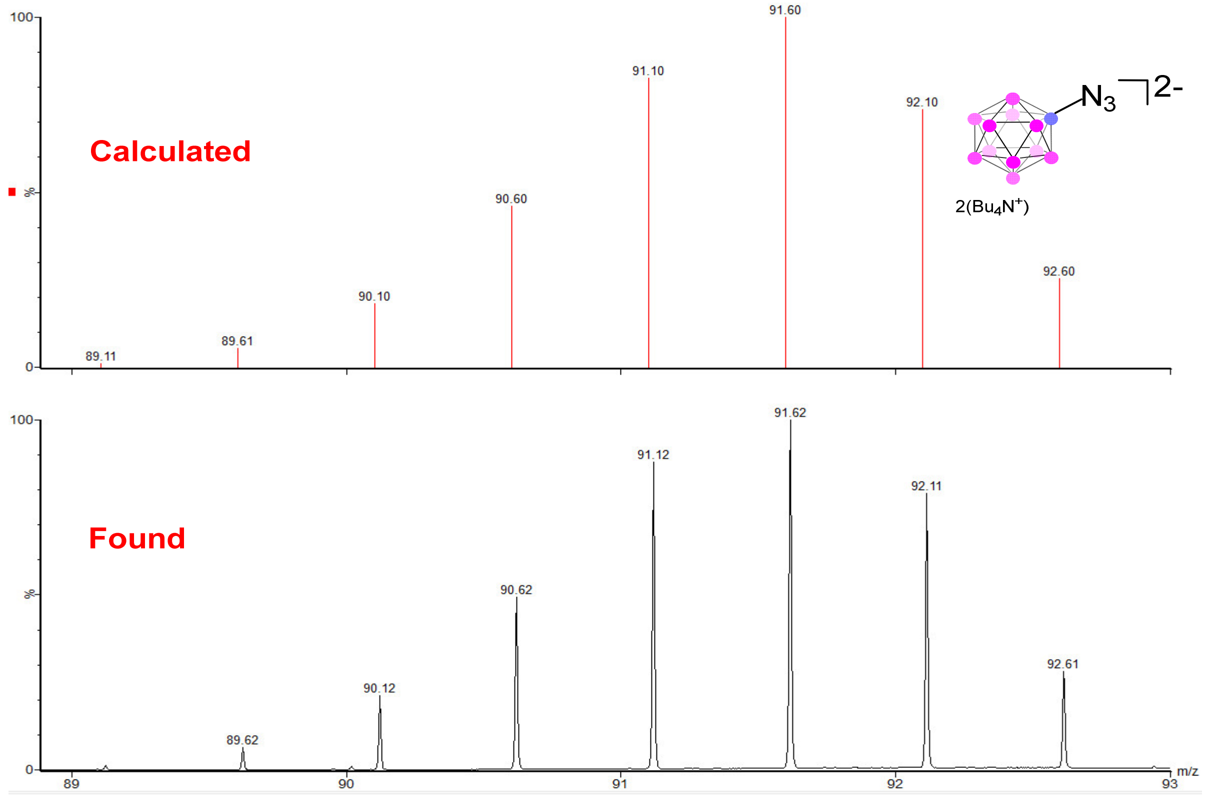

Figure S20. MS spectrum of $[B_{12}H_{11}N_3]^{2-}$.

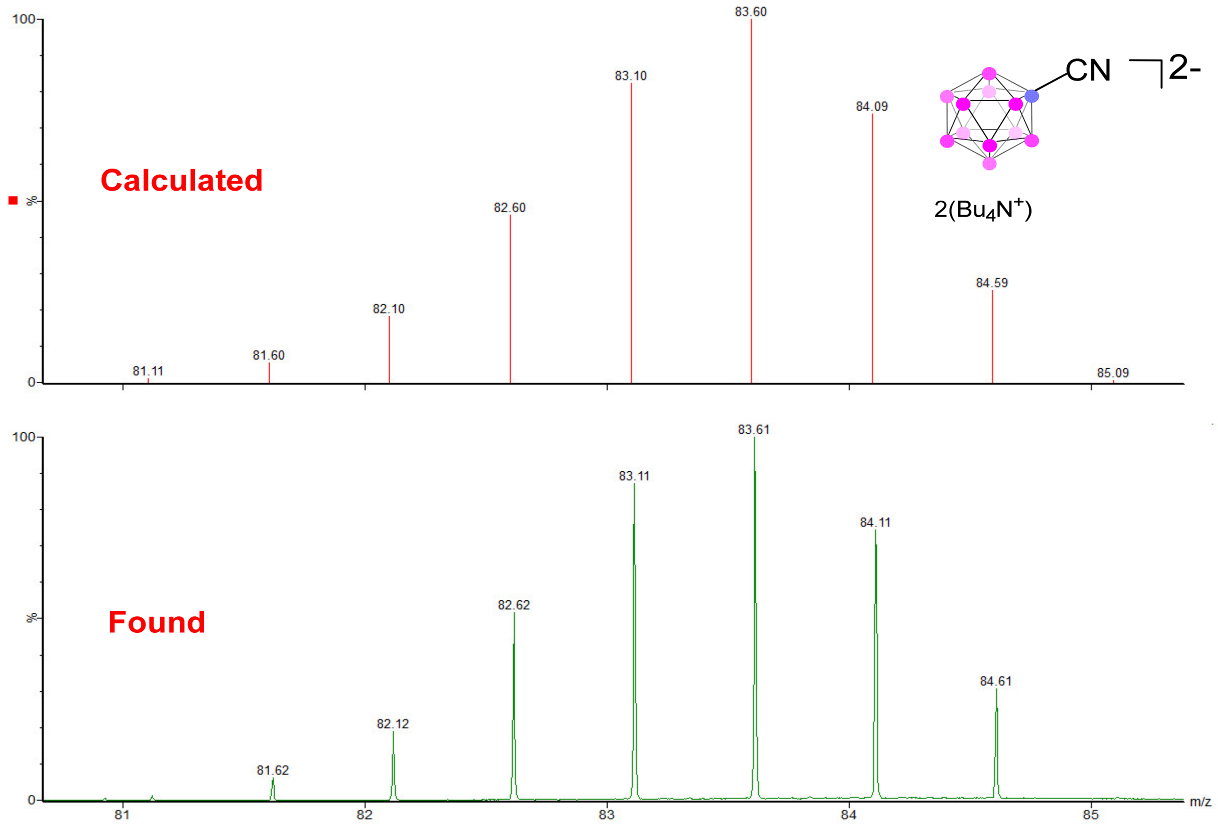

Figure S21. MS spectrum of $[B_{12}H_{11}CN]^{2-}$.

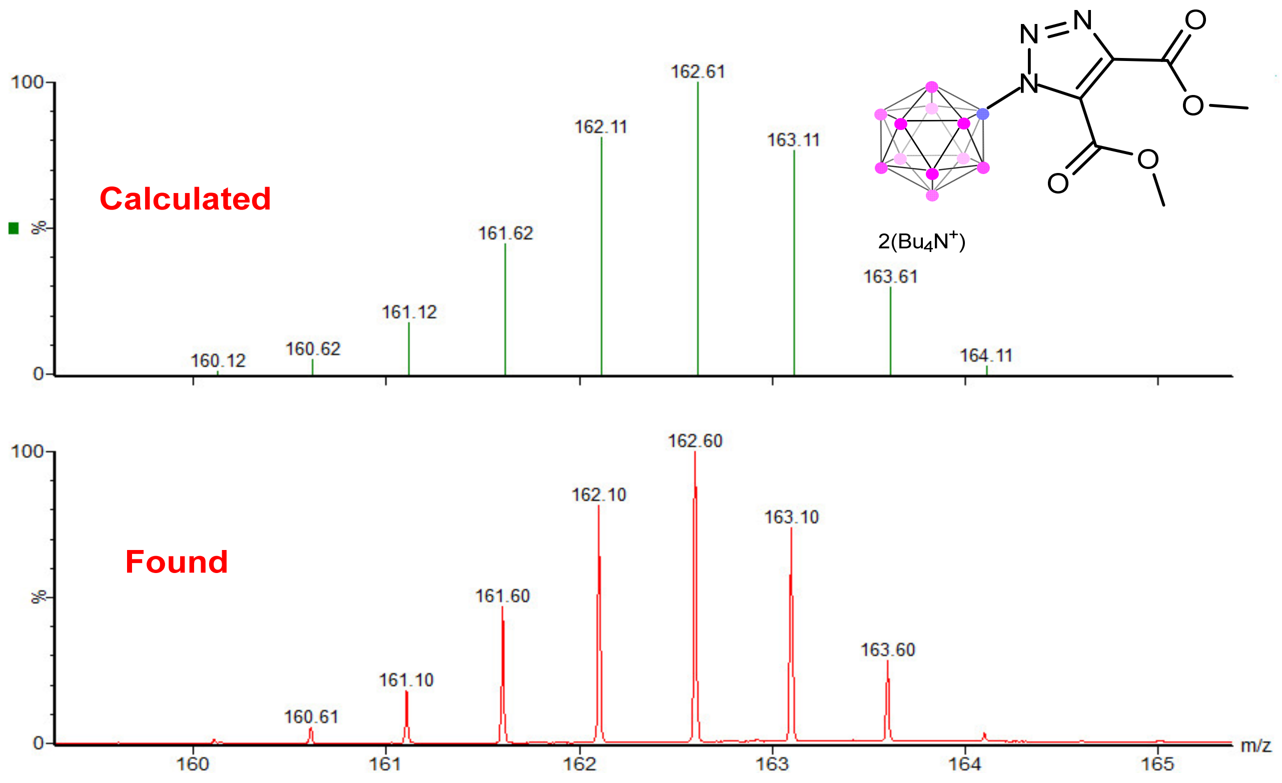

Figure S22. MS spectrum of $[B_{12}H_{11}\text{-}C_6H_6O_4]^{2-}$.
